# Who would most benefit from improved integrated care? Implementing an analytical strategy in South Somerset

**DOI:** 10.5334/ijic.1594

**Published:** 2015-01-28

**Authors:** Panagiotis Kasteridis, Andrew Street, Matthew Dolman, Lesley Gallier, Kevin Hudson, Jeremy Martin, Ian Wyer

**Affiliations:** Centre for Health Economics, University of York, UK; Health Economics, Centre for Health Economics, University of York, UK; Clinical Operations Group, Somerset Clinical Commissioning Group, Yeovil, Somerset, UK; South West Commissioning Support, Bridgwater, UK; Business Solutions and Innovation, South West Commissioning Support, Bridgwater, UK; The Symphony Project, Yeovil District Hospital NHS Foundation Trust, Higher Kingston, Yeovil, Somerset, UK; South Somerset Healthcare Federation, Milborne Port Surgery, Gainsborough, Milborne Port, UK

**Keywords:** integrated care, health and social care costs, morbidity profiles, capitated budgets

## Abstract

**Aims:**

The Symphony Project is designed to identify which groups of the South Somerset population in England would most benefit from greater integration across primary, community, acute and social care settings.

**Methods:**

We analysed linked health and social care data for the entire South Somerset population for the financial year 2012/2013. The data captured acute, primary, community, mental health and social care utilisation and costs; demographic characteristics; and indicators of morbidity for each individual. We employed generalized linear models to analyse variation in annual health and social care costs for all 114,874 members of the South Somerset population and for 1458 individuals with three or more selected chronic conditions.

**Results:**

We found that multi-morbidity, not age, was the key driver of health and social care costs. Moreover, the number of chronic conditions is as useful as information about specific conditions at predicting costs. We are able to explain 7% of the variation in total annual costs for population as a whole, and 14% of the variation for those with three or more conditions. We are best able to explain primary care costs, but explanatory power is poor for mental health and social care costs.

**Conclusions:**

The linked dataset makes it possible to understand existing patterns of health and social care utilisation and to analyse variation in annual costs, for the whole population and for sub-groups, in total and by setting. This has made it possible to identify who would most benefit from improved integrated care and to calculate capitated budgets to support financial integration.

## Introduction

Since the inception of the NHS, an ever-present challenge has been to improve integration of care within the health care system and with social care [1]. Many people have complex and ongoing care needs and require support from multiple agencies and various professionals [2]. But care is often fragmented and uncoordinated, with no one agency taking overall responsibility, so it is often left to individuals and their families to negotiate the system as best they can [3].

Traditionally, in England, health and social care funds have been channelled to institutions not individuals. In many instances, institutionally based funding fails to recognise that many people, particularly those with combinations of conditions, move across institutions, receiving care in multiple settings. But this creates problems. Patients find it difficult to negotiate their way through the health and care system. Care providers have had little financial incentive and have lacked financial mechanisms to allow funding to follow patients as they move from one setting to another. It has been recognised that financial arrangements need to be revised so as to support rather than inhibit organisations to work collaboratively around the needs of patients [4].

Over the last 15 years, the English government has taken several steps to improve the integration of health and social care in England. Statutory measures include the financial flexibilities (e.g. for pooling health and social care funds) first introduced in the 1999 Health Act and the introduction of Care Trusts which combined health and social care responsibilities within single NHS organisations. More recently, the Better Care Fund (formerly, the Integration Transformation Fund) has been proposed to support radical changes across the health and social care system by pooling funds of £3.8 billion [5], though this has proved controversial [6]. There have also been experimental projects, such as the Integrated Care Pilots (ICPs), the Partnerships for Older People Projects pilots and various local initiatives [7,8].

In a similar vein, in South Somerset, the county council, district hospital, community provider and clinical commissioning group have set up the Symphony Project to develop a tailored package of integrated care, centred on the needs of individual patients, particularly those with complex conditions. Somerset has a history of joint working; indeed, the Somerset Health and Social Care Trust was the first care trust to be set up in England [9]. Extending this tradition, the Symphony Project is designed to improve collaboration across primary, community, mental health, acute and social care. Collaborative working recognises joint responsibility by all organisations to deliver shared outcomes and is to be incentivised by linked financial arrangements.

To be able to realise these ambitions, the Symphony Project needs to be targeted initially at a subset of the population who would be expected to benefit most from integrated care. In what follows, we describe the process adopted to identify this initial group.

## Data and methods

The analytical objective of the Symphony Project was to identify which people might comprise the initial group to which integrated care arrangements would apply. Various methods to identify eligible groups have been used in other contexts. For example, the ICPs used methods including risk profiling tools and clinical judgement to target those at higher risk of hospital unplanned admission (Norfolk); presence in local general practitioners’ (GPs) dementia register (Newquay); reviewing of medical records to identify patients at risk of falling (North Tyneside); and identification of patients with moderate or severe chronic obstructive pulmonary disease (COPD) based on past hospitalisation or exacerbations of poor lung function (Northumbria) [10]. In the USA, Medicaid eligibility criteria and criteria for nursing home admission – e.g. significant functional problems and several chronic conditions – have been used [11].

We developed four broad criteria to identify groups most suitable for pooled funding arrangements that might facilitate integrated care [12]. First, to support financial integration, the number in the group needs to form a sufficiently large and stable “risk pool”. This should allow clear identification of who belongs to the group and ensure that those with high costs are offset by those with low costs. Second, to realise savings, initial focus should be directed towards those patients who are expected to be the highest users of services. We base expectations on current patterns of activity and cost [13,14]. Third, those using services across diverse settings are more likely to benefit from integrated care, simply because they are currently more likely to be subject to fragmented provision and duplication. Fourth, there should be local consensus that changes to the care pathway are feasible.

We assessed the first three of these criteria by examining patterns of health and social care utilisation and costs for South Somerset's entire population of 114,874 people in the financial year from March 2012 to April 2013. The Symphony Project has built a large data set comprising information about each anonymised individual in the South Somerset population. The data set has four key features: (i) it links acute, primary care, community, mental health and social care utilisation and activity data at individual level; (ii) costs are assigned to each individual according to the type of care they have received in each setting; (iii) demographic characteristics are available for each individual, including age, gender and socio-economic indicators; and (iv) critically, the data set includes indicators of morbidity for each individual.

### Describing morbidity

Each individual's morbidity profile is constructed using United Health's RISC tool [15]. RISC is a patient-level predictive modelling tool developed by United Health, UK, to assess the risk of patients having unplanned hospital admissions within a 12-month period. The tool utilises diagnostic information, described using ICD10 [16] and Read codes [17], in the patient's GP and hospital records. Chronic conditions are identified if the relevant diagnostic information appears in their GP record at any time in their medical history. The diagnostic information is summarised into 586 Episode Treatment Groups (ETGs) and 15 priority conditions, which are listed in the spreadsheet in the online appendix. Forty-nine of these ETGs and priority conditions are indicative of chronic conditions, and these formed the basis for describing the morbidity profile of each individual in the population. Individuals can, of course, have multiple chronic conditions.

We presented details of multi-morbidity profiles for those with particular conditions at a workshop with local health and social care professionals and managers in South Somerset [12]. Following this workshop, further analyses were conducted for those with at least three of a limited set of eight chronic conditions that local GPs viewed as most significant. These conditions were as follows: diabetes, cardiac disease, COPD or OPD, chronic kidney disease (CKD) or renal failure, depression or anxiety, dementia, stroke and cancer. In what follows we refer to the group with three or more of these conditions as the 3+ group.

### Utilisation and costs

For each member of the population, utilisation and costs were described in each setting of care. Primary care captures patient interaction with their general practice, including face-to-face contacts, telephone consultations and prescribing. Total primary care costs were allocated to each patient on the basis of the number of contacts they had during the year. Prescribing utilisation was captured by the RISC tool with the total local prescribing budget allocated to individuals according to the number of unique prescriptions received during the year. Acute and community care utilisation was based on activity information sourced through the NHS Secondary Uses Service including the number, type and length of episodes. Costs were based on the national tariffs associated with the particular Healthcare Resource Group [18] to which each patient was assigned. Mental health utilisation was based on nationally defined ‘mental health clusters’ [19], with costs calculated by Somerset Partnership, the local provider of mental health services. Social care utilisation was based on activity and cost information provided by Somerset County Council, including utilisation of home care, residential placements, day care, professional services, direct support and provision of equipment. Continuing care was based on activity and cost information provided by the local Continuing Healthcare team.

### Analytical model

We applied multiple regression models to analyse each person's total costs and costs incurred in each of the eight settings. We conducted the analysis for the whole population and for the 3+ group.

While most people had some form of primary care contact during the year, many people utilised no care in inpatient, outpatient, accident and emergency (A&E), mental health, community care, social care and continuing care settings. Consequently, to analyse costs in such settings, we employ two-part models [20–22] which allowed us to account for the large number of zeros found in the data. The two parts are assumed to be independent and can be estimated separately. The first part, estimated by a logistic regression, models the probability of incurring any expenditure, and the second part models the amount of expenditure only for those with positive costs. Primary care costs and total costs for the 3+ group exhibit a very small proportion of zeros and are estimated with a single model.

To model positive costs, we compared a generalised linear model (GLM) with log link and gamma distributed errors [23–25] with a log-linear regression model where costs are logarithmically transformed to reduce skewness and normalise the distribution. When the error term in the log transformation model is not normal, the non-parametric Duan smearing estimator [20] is usually applied under the assumption of homoscedasticity: the exponentiated linear predictor is multiplied by a smearing factor which is calculated as the average of the exponentiated least squares residuals.

We compare GLM and log-linear model performance in terms of *R*^2^ (of the regression of costs on predicted costs on the raw scale), Root Mean Square Error, and Mean Absolute Prediction Error. As shown in Table 1, the GLM model performed better in all settings with the exception of mental health, so we focus on these GLM results in what follows.

**Figure 1.**
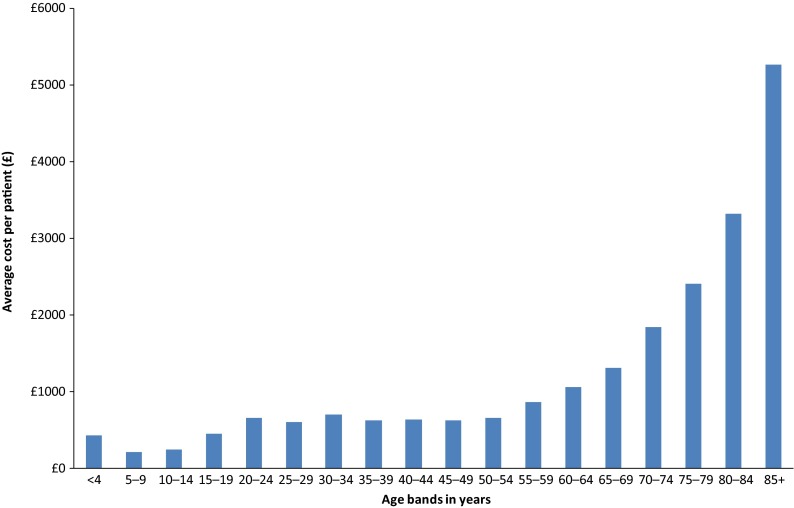
Average costs by age group.

Our set of explanatory variables included gender, the percentage of people in income deprivation in the local area, indicators for whether the individual died during the course of the year or moved elsewhere, the number (and square) of all chronic conditions, eight binary comorbidity variables indicating whether or not a person has one of the limited set of eight chronic conditions and two continuous age variables (age_p1 and age_p2) that capture the effects of age on costs for people younger than 55 and older than 55 years. This age specification is motivated by Figure 1 which plots average costs for the South Somerset population by 5-year age bands: age appears to be a piecewise relationship of costs with a segment for people younger than 55 years for whom costs vary little with age and another segment for people above 55 years old whose costs increase ever more steeply with age. As in other studies [13–22], we account for socio-economic circumstances by using the income deprivation domain of the English Index of Multiple Deprivation (IMD 2010), which measures the proportion of the population in each person's residential area that lives in income-deprived families. We use the data available at Lower Super Output Area (LSOA) level – the smallest geographical area in England for which deprivation information is collected [26].

## Results

Table 2 summarises the characteristics of the whole Somerset population of 114,874 people (columns 1 and 2) and of the subset of 1458 people with three or more of the eight chronic conditions, namely the 3+ group (columns 3 and 4). The 3+ group are more likely to be male and older (53% and 78 years) than the whole population (49% and 43 years). More than 11% of the 3+ group died during the course of the year compared to less than 1% in the whole population, and a smaller proportion (under 1%) moved out of the area (3% for the whole population). The percentage living in income deprivation is about the same in the two groups (10%). The average person in the 3+ group has 16 conditions (i.e. ETGs) and 5 of the 49 chronic conditions – the numbers for the whole population are 4 and 1.

Table 3 summarises annual utilisation and costs according to the type of health and social care for the South Somerset population as a whole (columns 1 and 2) and for the 3+ group (columns 3 and 4) for the full financial year 2012/2013. About two-thirds of the population and almost everyone in the 3+ group received prescribed medication. Almost four-fifths of the population and all but 4 patients in the 3+ group had a contact with their GP. Much smaller proportions of people in the 3+ group incurred costs in the other settings: 65% had an inpatient admission (15% in the population), 9% utilised community care (less than 2% in the population), 12% received mental health care (1.5% in the population), 31% had social care costs (less than 3% in the population) and about 9% had continuing care costs (just over 0.5% in the population).

For those who incurred costs in South Somerset, the average cost of care amounted to £1227, but there was wide variation in costs, with some people incurring high costs, reaching £17,830 at the 99th centile. The average total cost of care was significantly (*p* < 0.001) higher in the 3+ group at £8163. Average inpatient costs were also higher for the 3+ group reaching £4842 compared to £2549 for the population. Average annual costs for the 3+ group are significantly higher than for the general population in primary care, acute and community health care settings, but continuing care costs are lower (*p* = 0.04). Average annual mental health and social care costs are quite similar.

**Figure 2.**
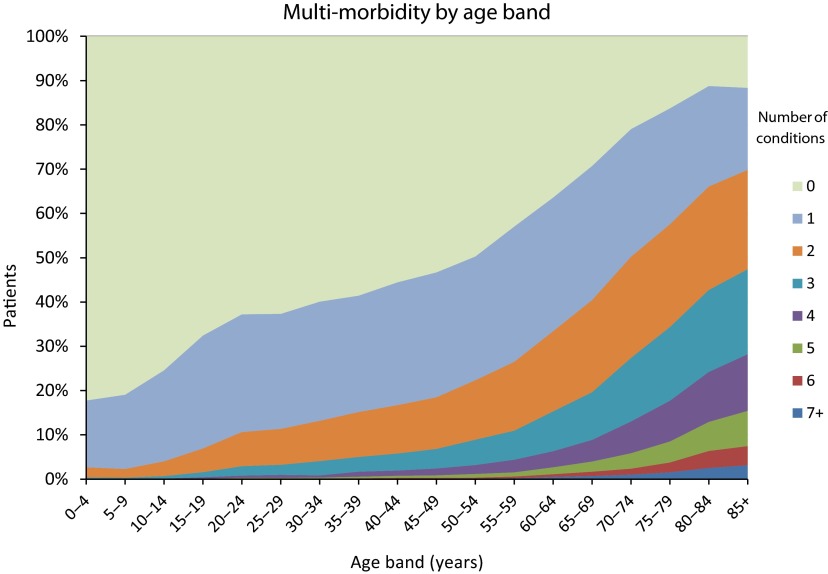
Relationship between age and the number of chronic conditions (ETGs).

The older that people are, the more the conditions they have. This is illustrated in Figure 2 which plots the number of chronic conditions by 5-year age bands for the full population. At the extremes, fewer than 20% of those aged 0–4 have 1 or more chronic conditions while almost 50% of those aged 85+ have three or more chronic conditions. We disentangled the relative contributions that age and the number of conditions have on costs. When we considered age by itself, it was able to explain only 6% of the variation in cost among the population. When we considered only the number of chronic conditions, these explained about 10% of the variation in costs among individuals. Including age alongside the number of chronic conditions added little explanatory power, *R*^2^ increasing by only 0.5%.

**Figure 3.**
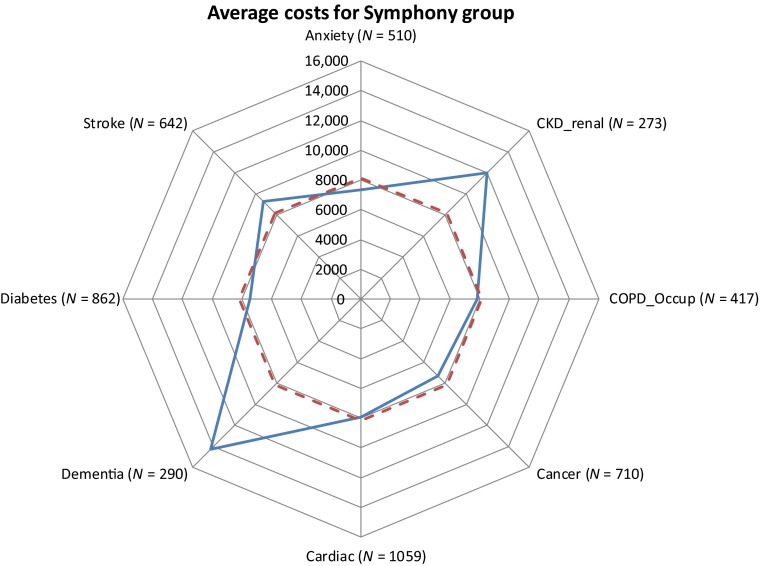
Average costs by co-morbidity for the 3+ conditions group.

Figure 3 shows the prevalence and costs associated with combinations of conditions among the 3+ group. The average annual cost of £8152 for all 1458 people in this group is indicated by the dashed circle. Costs vary from the average according to particular conditions. Most notably, costs are about £14,000 if dementia is among the conditions and £12,000 if CKD/renal is a co-morbidity, but there is little difference to the overall average for the other conditions.

We applied our GLM regression model to explain variation in positive costs (i.e. after excluding those with zero costs) in each setting for the full population (Table 4) and for the 3+ group (Table 5). Explanatory power, as indicated by the *R*^2^ from the regression of actual costs on the predicted scores on the raw scale, varies markedly by setting. The model explains 7% of variation in total costs for the full population and 14% for the 3+ group. Explanatory power is highest when explaining primary care costs, this being the setting in which most people were seen at least once during the year. At the other extreme, the model is able to explain very little of the variation in mental health care costs, whether for the full population or the 3+ group.

The specific factors that explain costs differ between the full population and the 3+ group and according to the setting under consideration. For the full population, costs increase significantly with age, but for the 3+ group age was positively associated only with community care costs for those under 55. In the general population, men have lower total, primary care and social care costs, but this is true only for social care costs among the 3+ group. Those among the whole population living in more deprived areas had higher total, primary care and A&E costs, but deprivation was not significantly associated with higher costs in other settings, nor in any setting for the 3+ group (except outpatient at *p* < 0.01). The number of chronic conditions is an important predictor of higher costs for the whole population in all settings except mental health, social and continuing care. The average person in the 3+ group had five chronic conditions, and increasing the number of conditions above this average explains higher costs only in primary care.

People who move into or out of the area during the year had higher primary care costs but lower outpatient and continuing care costs (Table 4), but migration is not significant in explaining costs among the 3+ group (Table 5). Those among the general population who died during the year had significantly higher costs in primary and acute care settings although continuing care costs are significantly lower. Among the 3+ group, those who died had higher total, primary care and inpatient costs, but lower outpatient and continuing care costs.

Whether someone had one of the eight specific conditions does not necessarily mean that their costs will be higher, with the coefficients often proving non-significant, particularly among the 3+ group (Table 5). For the whole population (Table 4), those with dementia had higher total annual costs than those without such diagnoses. For the 3+ group (Table 5), total annual costs were higher for those with CKD/renal and dementia. For CKD/renal, costs were higher in primary and inpatient settings; those with dementia had higher social care costs.

## Discussion and conclusion

The purpose of this study was to identify groups most suitable for pooled funding arrangements that might facilitate integrated care. Analysis was performed using linked health and social care data for the entire South Somerset population for the financial year 2012/2013. The data captured acute, primary care, community, mental health and social care utilisation and costs; demographic characteristics; and indicators of morbidity for each individual in the population. The analysis demonstrated that multi-morbidity, not age, was the key driver of health and social care costs. While costs were positively associated with age, accounting for age adds little explanatory power once we have accounted for the number of conditions in analysing costs.

The ability to measure morbidity is a significant advantage over studies that lack such information and are forced, instead, to rely on other variables to explain variation in costs. In their review of the literature, Zweifel et al. find that age and other socio-demographic information explain no more than 5% of the variation in health care costs; past utilisation increases explanatory power by around 1–2%, but the inclusion of information about morbidity adds more than 7% to explanatory power [27]. Similarly, Dixon et al. find that 12% of variation in costs is explained by morbidity characteristics [28]. Other studies have argued that proximity to death is a strong predictor of costs [29–32] but only in the absence of information about patient morbidity, which proves the more important explanatory factor [33].

In our study, we find that the number of chronic conditions that a person has is generally as important a predictor of costs as their specific conditions. This has been found previously in examining primary care costs [34], but we reach similar conclusions for costs in other health and social care settings. This is an important finding, because it reduces the information requirements for the design and calculation of capitated budgets to support integrated care, allowing budgets to be constructed in localities that are able to count the number of conditions a person has but that lack the detailed morbidity data available for the South Somerset population.

Integrated care is considered particularly important for the frail elderly [35], but identifying such people is challenging because of the difficultly in defining and measuring frailty using routine data [36,37]. Instead, the Symphony Project decided that the group of people most appropriate for integrated care arrangements should comprise those with three or more conditions from a set of eight chronic conditions deemed most important by local GPs. The main reasons for selecting this group were:

1. This group of around 1500 patients offered a reasonably high level of predictable cost variation, providing a sufficiently large risk pool for financial management;
2. The group incurred costs across all settings, thereby offering the prospect of strengthening links across health, mental health and social care;
3. There is an opportunity to reduce inpatient costs, which currently account for 38% of total costs for this group.

The ambition in South Somerset is to develop integrated care arrangements initially for the cohort of people 3+ chronic conditions, through the development of personalised care planning and supported self-management. The hope is then to extend arrangements to a larger cohort including all patients with long-term conditions.

The data set has been a key part of the foundations on which integrated care is being developed in South Somerset. In many parts of England, health and social care data are not brought together into a single data set and, in those areas where utilisation data have been joined together, information on costs and morbidity is lacking. The linked data have made it possible to understand existing patterns of health and social care utilisation and to analyse variation in annual costs, for the population as a whole and for members of the 3+ group, in total and by setting. In terms of each setting, our model performs best at explaining primary care costs, but explanatory power is poor when it comes to explaining mental health and social care costs, so further research is required to identify drivers of costs in these settings.

The data make it possible to calculate capitated budgets for different groups of the population, informed by current patterns of utilisation, and to use the model parameters to refine predictions of budgetary requirements, should the compositional characteristics of the group change or if there are changes across the settings in which care delivery takes place.

A drawback is that we have analysed only a single years’ worth of data, which means that we can identify only associations not causal relations between costs and the explanatory factors. This is true of all cross-sectional studies, although our data have the advantage that chronic conditions are identified if they have ever appeared in the patient's medical history, not just in the year in question.

The data are also from a relatively small locality, which limits generalisability of the results. However, the methods are generally applicable and, by demonstrating that the analytical approach can be applied to a small area, we believe that these methods can support local attempts to introduce integrated financial arrangements. Moreover, by maintaining the data set year on year, it will be possible to assess whether the efforts made to improve integrated care for the local population of South Somerset succeed in realising the ambition of the Symphony Project.

## Figures and Tables

**Table 1.**
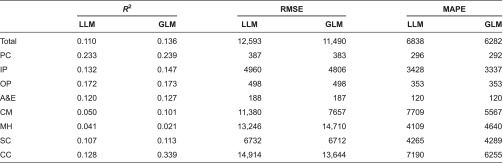
Measures of performance for the log-linear model (LLM) and generalised linear model (GLM) – 3+ group

PC, primary care; IP, inpatient; OP, outpatient: A&E, accident and emergency; CM, community care; MH, mental health care; SC, social care; CC, continuing care; RMSE, Root mean square error; MAPE, Mean absolute prediction error.

**Table 2.**
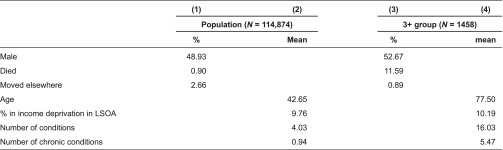
Summary statistics – background variables

**Table 3.**
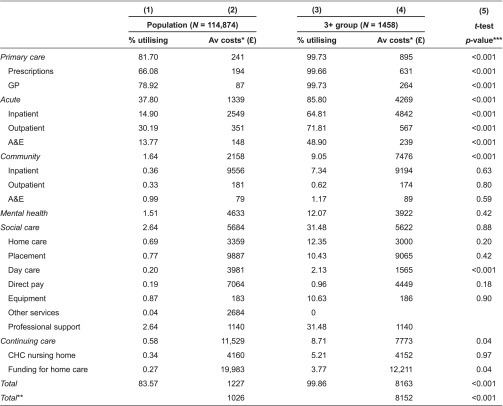
Summary statistics – utilisation per person and average costs (£), 2012–2013

*average costs calculated averaging over those with positive costs; **average total costs calculated averaging over the whole sample; ***p-values of independent *t*-tests comparing costs for those with positive costs in the 3+ group and the rest of population.

**Table 4.**
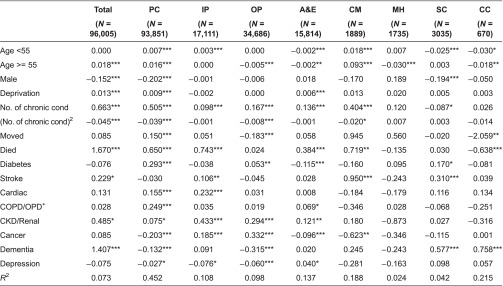
Influence of characteristics in explaining positive costs for the whole population

PC, primary care; IP, inpatient; OP, outpatient: A&E, accident and emergency; CM, community care; MH, mental health care; SC, social care; CC, continuing care

^+^COPD, chronic obstructive pulmonary disease; OPD, occupational pulmonary disease; CKD, chronic kidney disease.

*p < 0.05; **p < 0.01; ***p < 0.001.

**Table 5.**
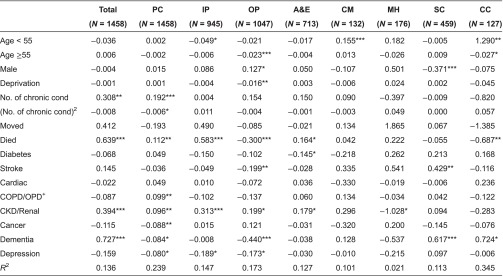
Influence of characteristics in explaining positive costs for those with 3+ conditions

PC, primary care; IP, inpatient; OP, outpatient: A&E, accident and emergency; CM, community care; MH, mental health care; SC, social care; CC, continuing care.

^+^COPD, chronic obstructive pulmonary disease; OPD, occupational pulmonary disease; CKD, chronic kidney disease.

*p < 0.05; **p < 0.01; ***p < 0.001.

## References

[1] Glasby J, Dickinson H, Miller R (2011). Partnership working in England: where we are now and where we've come from. International Journal of Integrated Care.

[2] Lehnert T, Heider D, Leicht H, Heinrich S, Corrieri S, Luppa M (2011). Review: health care utilization and costs of elderly persons with multiple chronic conditions. Medical Care Research and Review.

[3] Department of Health, National Collaboration for Integrated Care and Support (2013). Integrated care: our shared commitment. A framework that outlines ways to improve health and social care integration.

[4] Department of Health (2012). QIPP long term conditions: supporting the local implementation of the year of care funding model for people with longterm conditions.

[5] Local Government Association, NHS England

[6] The King's Fund. Better care fund, better read the small print?.

[7] Audit Commission (2008). The right result? Payment by results 2003–07.

[8] Mason A, Goddard M, Weatherly H (2014). Financial mechanisms for integrating funds for health and social care: an evidence review. CHE Research Paper.

[9] Ball R, Forbes T, Parris M, Forsyth L (2010). The evaluation of partnership working in the delivery of health and social care. Public Policy and Administration.

[10] National Evaluation of the Department of Health's Integrated Care Pilots (2012). RAND Europe and Ernst & Young LLP.

[11] Lynch M, Hernandez M, Estes C (2008). PACE: has it changed the chronic care paradigm?. Social Work in Public Health.

[12] Kasteridis P, Street A, Dolman M, Gallier L, Hudson K, Martin J (2014). The importance of multimorbidity in explaining utilisation and costs across health and social care settings: evidence from South Somerset's Symphony Project.

[13] Kadam UT, Uttley J, Jones PW, Iqbal Z (2013). Chronic disease multimorbidity transitions across healthcare interfaces and associated costs: a clinical-linkage database study. BMJ Open.

[14] Bardsley M, Georghiou T, Chassin L, Lewis G, Steventon A, Dixon J (2012). Overlap of hospital use and social care in older people in England. Journal of Health Services Research & Policy.

[15] HealthNumerics-RISK®.

[16] International Statistical Classification of Diseases and Related Health Problems 10th Revision.

[17] Health & Social Care Information Centre Read codes.

[18] Health & Social Care Information Centre Introduction to healthcare resource groups.

[19] Health & Social Care Information Centre Mental health care cluster.

[20] Duan N, Manning WG, Morris CM, Newhouse JP (1983). A comparison of alternative models for the demand of medical care. Journal of Business and Economic Statistics.

[21] Brilleman SL, Gravelle H, Hollinghurst S, Purdy S, Salisbury C, Windmeijer F (2012). Keep it simple? Predicting primary health care costs with measures of morbidity and multimorbidity.

[22] Charlton J, Rudisill C, Bhattarai N, Gulliford M (2013). Impact of deprivation on occurrence, outcomes and health care costs of people with multiple morbidity. Journal of Health Services Research & Policy.

[23] Blough D, Madden C, Hornbrook M (1999). Modeling risk using generalized linear models. Journal of Health Economics.

[24] Manning WG, J M (2001). Estimating log models: to transform or not to transform?. Journal of Health Economics.

[25] Manning WG, Basu A, J M (2005). Generalized modelling approaches to risk adjustment of skewed outcomes data. Journal of Health Economics.

[26] GOV.UK The English indices of deprivation.

[27] Zweifel P, Breyer F, Kifmann M (2009). Health economics.

[28] Dixon J, Smith P, Gravelle H, Martin S, Bardsley M, Rice N (2011). A person based formula for allocating commissioning funds to general practices in England: development of a statistical model. BMJ.

[29] Zweifel P, Felder S, Meiers M (1999). Ageing of population and health care expenditure: a red herring?. Health Economics.

[30] Werblow A, Felder S, Zweifel P (2007). Population ageing and health care expenditure: a school of ‘red herrings’?. Health Economics.

[31] Felder S, Werblow A, Zweifel P (2010). Do red herrings swim in circles? Controlling for the endogeneity of time to death. Journal of Health Economics.

[32] deMeijer C, Koopmanschap M, d'Uva T, van Doorslaer E (2011). Determinants of long term care spending: age, time to death or disability?. Journal of Health Economics.

[33] Howdon D, Rice N (2014). Age, proximity to death and ill-health, in the presence of compression of morbidity.

[34] Brilleman SL, Gravelle H, Hollinghurst S, Purdy S, Salisbury C, F W (2014). Keep it simple? Predicting primary health care costs with clinical morbidity measures. Journal of Health Economics.

[35] NHS England Safe, compassionate care for frail older people using an integrated care pathway: practical guidance for commissioners, providers and nursing, medical and allied health professional leaders.

[36] Chin A, Paw MJ, Dekker JM, Feskens EJM, Schouten EG, Kromhout D (1999). How to select a frail elderly population? A comparison of three working definitions. Journal of Clinical Epidemiology.

[37] Purser JL, Kuchibhatla MN, Fillenbaum GG, Harding T, Peterson ED, Alexander KP (2006). Identifying frailty in hospitalized older adults with significant coronary artery disease. Journal of the American Geriatrics Society.

